# Research on the cognitive neural mechanism of privacy empowerment illusion cues regarding comprehensibility and interpretability for privacy disclosures

**DOI:** 10.1038/s41598-024-58917-8

**Published:** 2024-04-15

**Authors:** Rui Sun, Qiuhua Zhu, Ru Xia Cheng, Wenlong Tang, Jiajia Zuo, Dong Lv, Shukun Qin

**Affiliations:** School of Business Administration, Huaqiao University, Quanzhou, 362000 China

**Keywords:** Cognitive neuroscience, Emotion, Social behaviour

## Abstract

In the era of artificial intelligence, privacy empowerment illusion has become a crucial means for digital enterprises and platforms to “manipulate” users and create an illusion of control. This topic has also become an urgent and pressing concern for current research. However, the existing studies are limited in terms of their perspectives and methodologies, making it challenging to fully explain why users express concerns about privacy empowerment illusion but repeatedly disclose their personal information. This study combines the associative-propositional evaluation model (APE) and cognitive load theory, using event-related potential (ERP) technology to investigate the underlying mechanisms of how the comprehensibility and interpretability of privacy empowerment illusion cues affect users’ immediate attitudes and privacy disclosure behaviours; these mechanisms are mediated by psychological processing and cognitive load differences. Behavioural research results indicate that in the context of privacy empowerment illusion cues with low comprehensibility, users are more inclined to disclose their private information when faced with high interpretability than they are when faced with low interpretability. EEG results show that in the context of privacy empowerment illusion cues with low comprehensibility, high interpretability induces greater P2 amplitudes than does low interpretability; low interpretability induces greater N2 amplitudes than does high interpretability. This study extends the scopes of the APE model and cognitive load theory in the field of privacy research, providing new insights into privacy attitudes. Doing so offers a valuable framework through which digital enterprises can gain a deeper understanding of users’ genuine privacy attitudes and immediate reactions under privacy empowerment illusion situations. This understanding can help increase user privacy protection and improve their overall online experience, making it highly relevant and beneficial.

## Introduction

In the era of digital intelligence, user information has gradually widened the inequality of information sovereignty in the field of data. Digital platforms have gained unprecedented control over user data. They no longer satisfy the real psychological needs of users, instead using a more covert way to control user data and give users an illusion of privacy control^1,2^. For instance, Meta has transferred European user data to servers located in the U.S. without clearly informing or seeking consent from Facebook users before doing so. Online users are instilled with the illusion of privacy control and become the data source of passive training algorithms, which ultimately only serve the interests of shareholders. For example, Google’s smart speakers further exploit this illusion of privacy, presenting a useful personalized façade while extracting private personal and family information, thereby depriving users of their informational autonomy and control without their knowledge. A report from Canada's “Defense and Security Innovation” (IDEaS) Center states that the illusion of privacy empowerment can not only influence public opinion but also affect the political process^3^. For example, the Brexit referendum and the US presidential election events that obtained Facebook user data without user permission led to the disclosure of private information without user knowledge.

To address the deep control of digital platforms over users through intelligent algorithmic technologies, countries have introduced corresponding laws and regulations. For example, in 2020, California passed an amendment to the California Consumer Privacy Act, and during the same period, Canada introduced the “Digital Charter Implementation Act of 2020”, among others. Subsequently, platforms also developed their own privacy policies to respond to the relevant laws and regulations. The privacy statements provided by digital platforms are often presented to users with vague, technical, and obscure terminology and are broad and ambiguous in nature^4^. While digital platforms fulfil the nominative duty of notification^5^, explicability and intelligibility are lacking; thus, these platforms fail to genuinely empower users with control over their privacy and merely give users the illusion of being empowered^6^. For instance, Facebook’s privacy terms provide explanations such as “manage your personal information” and “do not share data with third parties without authorization,” yet the data of 87 million Facebook users were collected without their consent for political purposes, stripping users of their privacy control rights. While some platforms provide certain privacy setting options, they are set to the sharing mode by default. Moreover, some internet platforms make very subtle changes to their confidentiality terms. These statements are filled with a plethora of legal jargon and overly broad language, leading to the illegal collection and sharing of user information. Digital platforms seem to give users privacy control on the surface, but in practice, they have not fulfilled their commitment to protect users’ privacy and ultimately achieve the purpose of implicitly collecting, analysing and using users’ private information. It is true that the infringement of user privacy by digital platforms has induced widespread dissatisfaction with the illusion of privacy empowerment, but users are still willing to accept the convenience of their own autonomy. An accurate analysis of the paradox of privacy empowerment can not only help digital platforms balance the problem of data governance with value rationality but also encourage governments to better carry out data governance and help enhance the precision of social governance. At the same time, it can prevent platforms from taking this paradox as a reasonable explanation for ignoring users’ privacy and security demands and ignoring the users’ real experiences, which result in reputation losses for the platforms and ultimately losses of users. Therefore, there is an urgent need to clarify the internal mechanism of the privacy empowerment paradox.

The existing research has encountered difficulty when attempting to fully explain the paradox of privacy empowerment due to the limitations of the utilized research perspectives and methods. The existing studies, which are mainly based on theories such as privacy calculus, communication management, and privacy cynicism^7–10^, somewhat explain the rationale behind individuals’ self-protective motives through rational analysis, assessing the overall threat of privacy empowerment illusion cues and thus leading to certain privacy actions^7,11, 12^. However, many related studies rely on retrospective situations because users’ real online privacy behaviours in immediate contexts are not the result of extensive rational analyses and are instead influenced by situational factors such as their cognitive loads. Attitudes constructed based on these immediate factors that reflect reality are effective at ultimately shaping individual behavioural decisions^13,14^. Concurrently, within immediate situations, users do not comprehend the abstract and ambiguous indicators of the privacy empowerment illusions they receive, leaving their privacy apprehensions unaltered; abstract, vague explanations consume significant cognitive resources such as attention and emotion, leading to cognitive overload; and variations in users' cognitive loads are key variables that influence individual behavioural decisions^15^^.^ However, little research has been conducted on users’ cognitive loads in immediate contexts and their instantaneously constructed views on the paradox concerning the privacy empowerment illusion, specifically, the internal mechanisms of users' privacy attitudes and privacy disclosure behaviours. Additionally, the prevailing research predominantly employs survey methodologies, but questionnaire results represent hypothetical responses to hypothetical situations and overall assessments that are retrospectively processed and analysed by individuals (“Only when you inquire, do I become aware?”). These assessments are influenced by recollection and personal subjective elements^16^, making it difficult to accurately reflect users’ cognitive processes under true privacy empowerment illusion situations in immediate contexts and preventing precise alignment between the cognitive loads experienced during decision-making contexts and during post hoc responses; these issues prevent the practical study of the state of an individual's instantaneous cognitive load.

To address the aforementioned issues, this study selects the most common privacy empowerment illusion cues—such as the comprehensibility and interpretability of the privacy statements contained in privacy policies and popup prompts—as research variables. It introduces cognitive load theory and the APE model and employs experimental brain neuroscience methods. This enables a precise understanding of the privacy empowerment paradox, offering in-depth insights into the underlying mechanisms by which individual privacy empowerment affects the immediate attitudes and privacy disclosure behaviours of individuals. Event-related potential (ERP) technology possesses a high temporal resolution at the millisecond level, allowing for accurate simulations of real-time privacy decision-making scenarios. Furthermore, this technology is considered a “magnifying glass” for observing psychological processes, as it can pinpoint individual cognitive processes without needing to directly inquire about user thoughts, memories, evaluations, or decision strategies^17^. It is less influenced by subjective individual interferences and offers more scientific and precise data representations of individuals' cognitive processes and immediate responses in online contexts. This approach aids in unravelling the puzzle of “users expressing significant concerns about privacy empowerment illusion but remaining apathetic toward privacy protection.”

## Review of the relevant research

### Privacy empowerment illusion

The concept of empowerment originated in sociology and psychology and stems from individuals’ inherent need for autonomy. Self-efficacy pertains to “empowerment” or “self-efficacy,” signifying the process by which people increase their awareness of personal efficacy, increase their motivation to achieve goals, and consequently experience control over their circumstances^18^. Empowerment theory focuses on providing more opportunities and resources for socially disadvantaged groups^19^ to help them gain greater power, reach higher statuses and realize their potential; this idea is fundamentally aimed at diminishing the sense of powerlessness within disadvantaged groups^20^ and enhancing their confidence and agency. This theory is typically employed to aid groups such as those facing poverty and disability, such as by legally providing people with disabilities with accessible environments and job opportunities to help them integrate into society and providing women with equal political rights, thus enabling them to possess the same societal status as men, among others. All these actions embody the fundamental values of empowerment theory, that is, elevating the agency and statuses of vulnerable populations. In the era of big data, the concept of empowerment has shifted from traditional interactions among individuals, organizations, and entities to relationships between digital platforms and users. However, despite this shift in focus, the most fundamental issue that empowerment theory aims to address remains unchanged: empowering "vulnerable groups." In the process of providing power to users through digital platforms, platforms often have relatively strong advantages over data sovereignty, while users are relatively weak and controlled within a relatively limited range.

Privacy empowerment illusion refers to a platform giving users the power to manage their privacy, allowing the users to perceive control; however, this opportunity is not effective and merely serves to create an illusion of empowerment^6^. The existing related research has focused primarily on two aspects. First, the effectiveness of privacy protection methods for users in the context of data monopolies has been explored. Most existing privacy protection methods are based on individual control and choice and often overlook the immense power and influence of certain digital platforms^2^. In the monopolistic realm of big data economics, true choices are seldom provided to users, as they are manipulated into the idea of compromising their autonomy through choices, leading to the acquisition of personal information. Digital platforms control the scope of information users access online and can steer users towards choices that are favourable to the digital agendas of the platforms. Based on the relevant privacy empowerment statements, the notification and selection mechanism is only used to help digital platforms shift people's attention to their responsibilities^21^. On a personal level, users find themselves unable to resolve the dilemma between their need for participation and connection and the need to protect their privacy^22^, leaving them with the option of relinquishing their participation rights in the data world, becoming isolated, or consenting to their data being sold. Second, negative perceptions and behaviours arise when users feel that they have lost control over their data rights due to privacy empowerment illusion. Some scholars have argued that inappropriate practices, such as abusing private data, monitoring users, and tracking users based on privacy empowerment, strip users of their data sovereignty. This leads to users having negative perceptions, including senses of lost control, perceived threats, and psychological resistance, which can trigger negative emotions and subsequently have a detrimental impact on users' privacy-related behaviours^23^. Additionally, some researchers have suggested that as privacy breaches continue to occur, users may perceive privacy infringements as inevitable and feel that they have lost control over their personal data^24^. As a result, they may experience privacy fatigue and adopt a passive attitude^25,26^, leading them to disclose their personal data even in situations involving privacy empowerment illusion.

### Privacy empowerment illusion cues and privacy disclosure

By examining the relevant literature, it is found that the most common way that digital platforms give users the illusion of privacy empowerment is to use privacy policies and notification pop-ups as privacy statement cues. For example, users are immediately prompted to read privacy agreements in the form of pop-ups and full-screen pages upon first launching the QQ music app; if the users do not agree, they are reminded again via pop-up windows, such as when opening the Starbucks app, after which the privacy policy is announced via a “pop-up” reminder on the homepage. These practices engender an illusion of empowerment among users, leading them to believe that they possess autonomy and control. In the study of cues related to privacy empowerment illusion, the pertinent discussions conducted by scholars can be summarized from the following perspectives.

In terms of presentation, scholars have analysed the impact of the observability of privacy empowerment illusions in privacy statements on user privacy concerns. When privacy empowerment illusions in privacy statements occur in locations on webpages or websites that are more noticeable or prominent, they significantly increase the attention paid by users to private information and increase the importance of this information^27^. Several scholars have found that the public declaration of data usage to target users through AdChoices icons leads users to perceive brands as trustworthy. This makes it more challenging for users to identify and interpret the persuasive elements of privacy empowerment illusion on digital platforms, thereby making them more willing to grant privacy permission and accept personalized information recommendations^28^. Aguirre and colleagues found that, compared to implicit data collection from users, declarations about the public collection of private user information are more likely to increase users’ senses of privacy control, significantly reduce their perception of privacy risks, and increase their willingness to disclose private information^29^. Furthermore, some scholars have investigated the impact of the length of privacy statements on user privacy concerns. They found that participants who saw shorter policies spent less time reading but had a greater understanding of social media privacy practices due to their longer per-word reading times^30^.

Regarding the comprehensiveness of information content, scholars often claim that enhancing the comprehensiveness of privacy statements can effectively alleviate user concerns about privacy. The use of statements related to privacy empowerment can effectively increase the effect of empowerment illusion on users and increase their self-efficacy in terms of privacy control, resulting in privacy leakage. Some researchers have found that the contents of current privacy statements remain incomplete, with digital platforms displaying at most one attribute for each piece of information (such as location information or storage information). This suggests that they disclose only partial attributes of the use of private information and that their privacy statements are incomplete^31^. On the basis of qualitative research, some scholars have arrived at the same conclusion, namely, that the existing explanations of privacy statements are incomplete. Users wish to receive genuine privacy empowerment and seek explanations regarding the specific details of how digital enterprises and platforms collect, use, and analyse their data. They do not want the mere illusion of empowerment, which might lead them to mistakenly believe that they possess sovereignty over their privacy^32^.

However, scholars have expressed doubts about the effectiveness of privacy statements under digital monopolies. On the one hand, some scholars have claimed that such privacy statements are effective. Research has shown that, compared with companies that do not provide privacy statements, companies with privacy statements exhibit increased transparency in terms of their use of private data, which can effectively alleviate user perceptions of risk and increase their trust in these companies^33^. Moreover, privacy statements can effectively increase users' sense of control over their own private data. The more explicit an informative statement is, the stronger the users' sense of control over their own privacy, making them more inclined to disclose their own private information and react positively to digital platforms^34^. On the other hand, some scholars believe that the privacy statements given by digital platforms are more like disclaimers, making users more likely to have a sense of resistance to the illusion of empowerment^35^. Based on the perspective of technological threat avoidance, some scholars have found that higher-level privacy statements significantly increase users’ ability to perceive illusions of control and threats, while threat perception causes users to make negative behavioural decisions^5^. Kim et al. also showed through an experimental study that when users discover that platforms excessively collect and use their personal information, their sense of control diminishes, and their concerns about their own privacy outweigh their preference for personalized services^36^.

In summary, the existing studies are based on theories such as privacy calculus theory and communication privacy management^7–10^, which influence behavioural intentions from the perspective of users as rational beings. However, what users perceive is often retrospective and based on rational analyses, which may not always align with their immediate online decision-making behaviours. Most users construct their immediate attitudes and subsequently make decisions based on immediate emotions and the cognitive overload triggered by privacy statements, which is associated with false empowerment. The internal differences among users' immediately constructed privacy attitudes and cognitive loads play a crucial role in determining their behavioural intentions. However, limited research has been conducted from the perspectives of the psychological loads and immediate constructions of users to explore the impact of privacy empowerment illusion clues on privacy disclosure. How do the interpretability and comprehensibility of privacy empowerment illusion clues affect user privacy disclosure mechanisms? What kinds of cognitive loads do users generate to construct immediate attitudes? Considering the internal effects of the interpretability and comprehensibility of privacy empowerment illusion clues on user privacy disclosure, users' perceptions of the interpretability and comprehensibility of privacy empowerment illusion clues are based on differences among the cognitive resources invested during processing. Therefore, this study intends to investigate the impact of the interpretability and comprehensibility of privacy empowerment illusion clues on privacy disclosure from the perspectives of the cognitive loads and immediate constructions of users by using cognitive load theory and the APE model. The present study utilizes experimental ERP technology to analyse the underlying cognitive mechanisms in both behavioural and experimental EEG data.

## Theoretical basis and research hypothesis

### Theoretical basis

#### APE model

The construction of attitude concepts in the associative-propositional evaluation model (APE) is based on two cognitive psychological processes: associative processing and propositional processing^37^. Associative processing is defined as the activation of associations in memory based on the features presented by external stimuli and the available memory, and this activation is driven by pre-existing similarity cognitions. In contrast, propositional processing is defined as the activation of information implied by activated associations. It is assumed to be guided by the principle of logical consistency^38^. The psychological procedure of associative processing involves the activation of associations stored in memory and is generated through an associative evaluation process. The most prominent features of the associative evaluation process are the automatic emotional responses of individuals to specific stimuli, which are independent of ground-truth values and unrelated to subjective notions of right or wrong^37,39^. Pattern activation involves matching previously stored associative structures in memory with specific external stimuli in a given context and subsequently activating specific associations. In contrast, the latent psychological procedure of propositional processing is represented as evaluative judgements guided by deductive reasoning; this strategy is dependent on ground-truth values and is related to subjective judgements of right and wrong. During propositional processing, the automatic emotional responses generated during associative processing enter the thinking system and produce corresponding propositions^40^. Furthermore, the APE model introduces interactions between associative processing and propositional processing, encompassing the impact of associations on propositions, the impact of propositions on associations, and the collective impact of associations and propositions on behaviour. Through these assumptions, the APE model can explain and predict how people's evaluations of things are generated, how they change, and how they are expressed in various situations.

### Cognitive load theory

Cognition is the ability to acquire and process information during the problem-solving process^41^. In 1988, the psychologist Sweller introduced cognitive load theory, which posits that cognitive load represents the total cognitive resources consumed by the cognitive system during information processing in the context of a specific task. It reflects the “mental effort” individuals must exert during information processing while undertaking a task^42^. Cognitive load theory is grounded in the human cognitive structure and posits that humans have a finite working memory capacity. When the information demands of a cognitive task surpass this capacity, cognitive overload occurs; conversely, cognitive underload occurs in other situations^43^.

According to cognitive load theory, cognitive loads can be divided into intrinsic cognitive loads, extraneous cognitive loads, and associated cognitive loads^44^. Intrinsic cognitive loads are related to the complexity of information or tasks, with more difficult tasks imposing greater loads on working memory. An extraneous cognitive load occurs due to an inappropriate task presentation, leading to unnecessary cognitive operations in individuals, thereby subjecting their working memory to a certain load. When the presentation of a task is unfavourable for cognitive processing, the cognitive construction process of the individual is hindered, resulting in a greater extraneous cognitive load; conversely, a lower extraneous cognitive load occurs in other scenarios. An associated cognitive load is a load related to facilitating schema construction and automation during the learning process and is typically associated with knowledge acquisition. Several studies have suggested that cognitive loads, as factors affecting individuals' cognition, are playing an increasingly important role in understanding the psychology and behaviours of individuals in the information age^45,46^. For instance, in online shopping cases, research has examined the behavioural intentions of recommender system users and found that the complexity of product presentations and website pages can impact users' emotions and cognitive processes. When the complexity level is too high, it can lead to emotional and cognitive overload, resulting in negative effects^45^.

### Research hypothesis

#### Behavioural assumption

Digital platforms often use privacy cues such as privacy policies and pop-up notification messages to inform users about how their information will be used, i.e., collecting and using personal information in an accessible, explainable, and understandable manner^47^. This is done to increase users’ potential sense of control over their data^7^, prompting users to overlook the risks associated with privacy empowerment illusion on digital platforms. Previous studies often categorized privacy empowerment illusion statements into three dimensions: perceived information disclosure, perceived clarity, and perceived accuracy^12,48^. Perceived information disclosure is the extent to which information is made public and explainable, i.e., the interpretability of privacy empowerment illusion cues. Perceived clarity is the degree to which information is understood rather than considered vagueness, i.e., the comprehensibility of privacy empowerment illusion cues. Perceived accuracy represents whether the given information reflects reality rather than being exaggerated or biased, i.e., the truthfulness of empowerment cues. the existing research has focused mostly on the impact of the comprehensibility of privacy statements on user privacy concerns. Research suggests that users spend less time on privacy statements that are easy to understand, simple, and highly comprehensible but that they gain a better understanding of privacy practices on social networking sites. Furthermore, simple and comprehensible privacy policies increase users’ trust in platforms^30^, but they often lack detailed information^49^ and hinder users’ informed consent and decision-making processes^48^.

Based on the different comprehensibility levels of privacy empowerment illusion cues, the levels of detail in their explanations have differentiated impacts on user behavioural responses^50,51^. Low comprehensibility occurs when abstract, relatively holistic characteristics, such as concepts based on industry terminology and complex mathematical symbols, are used to represent information. In contrast, high comprehensibility occurs when more precise and specific information representations are provided. Faced with privacy empowerment illusion cues possessing high comprehensibility, users can clearly understand how recommendation systems collect and use their personal information, which not only generates a sense of trust but also mitigates the negative emotions associated with privacy infringement, reducing the inner worries and uncertainties of the users and alleviating their anxiety related to data leakage and misuse^6^. However, digital platforms often provide information with low comprehensibility that is difficult to understand and vague and lacks explanatory power not only to create a sense of trust but also to mitigate the negative emotions associated with privacy infringement, reducing users’ inner worries and uncertainties and alleviating their anxiety related to data leakage and misuse^45^. Such low-comprehensibility information also requires users to expend more cognitive resources when processing it^30,45^.

Due to the limited cognitive resources and capabilities of users, when low-comprehensibility privacy empowerment illusion cues have more concise, clear, and specific information elements, individuals need to expend fewer cognitive resources, resulting in less cognitive effort being needed. According to attitude construction theory, an individual's current cognitive state is a determining factor in successfully inhibiting automated attitudes in evaluative judgement scenarios^52,53^. In the context of privacy empowerment illusion on digital platforms, users have long been aware of the harm caused by platform manipulation but still implement few privacy protection measures. This is because users' attitudes towards privacy empowerment illusions on platforms are mostly constructed immediately and are highly influenced by situational cues, such as the explanation levels of privacy statements. When privacy empowerment illusion statements are clearer and more specific, individuals are more likely to activate past memories through associative processing and to construct attitudes based on perceptual cues without expending excessive cognitive effort. In other words, individuals are more likely to disregard the harm caused by privacy empowerment illusion and subsequently exhibit positive behavioural responses when provided with “high-explanatory” cues from a platform^54^. When privacy empowerment illusion statements are vague and abstract and the situational cues become more complex, individuals find it difficult to directly activate useful cues from past memories through associative processing for decision-making purposes. When individuals’ attitudes become less clear, they tend to engage in the proposition-based processing of vague, abstract information, which consumes more cognitive resources. Attitudes constructed under cognitive overload negatively impact user behaviours^45^. Due to the limited availability of cognitive resources, when current tasks require individuals to expend a significant amount of cognitive resources, they tend to exhibit more resistance based on the attitudes they construct in real time according to situational cues to ensure efficient cognitive processing of objective matters. Thus, this study proposes the following hypotheses.

##### H1

When privacy empowerment illusion cues with low comprehensibility are presented, users are more inclined to disclose their private information in response to high-interpretability cues than in response to low-interpretability cues.

##### H2

When privacy empowerment illusion cues with high comprehensibility are presented to users, interpretability does not significantly affect users’ privacy disclosure behaviours.

### ERP hypothesis

P2 is a positive component with a latency of approximately 200 ms and is primarily activated in the frontal and parieto-occipital regions of the brain. P2 is considered related to psychological processes such as users’ attentional biases and emotions and thus can reflect users’ attention, preferences, and emotions. This study primarily investigates the extent to which the P2 component arouses users' negative emotions. Several studies have indicated that the amplitude and latency of P2 can reflect the arousal of users' negative emotions and their preferences for stimulus cues^55^; for example, during emotion induction tasks, the P2 amplitude is affected by the emotional valence and intensity levels when users react to positive or negative emotional stimuli^56^. Ito and colleagues found that stimuli with negative valences cause significant changes in the P2 component, with significantly larger amplitudes in highly negative emotional contexts than in lowly negative emotional contexts^57^. Many scholars claim that stimuli with negative valences elicit larger P2 amplitudes than stimuli with positive valences^55^. During preference-choice tasks, consumers' reactions to options they like or dislike affect the amplitude of P2, which is influenced by their preference and consistency degrees^58^. When presented with privacy empowerment illusion cues possessing high comprehensibility, individuals with clear attitudes do not make decisions based on situational cues, resulting in low decision uncertainty. Therefore, the P2 amplitudes induced by high- and low-interpretability cues do not exhibit significant differences. However, when presented with privacy empowerment illusion cues possessing low comprehensibility, individuals struggle to extract meaningful information directly from situational cues. Instead, individuals rely on comprehensibility cues, and individuals find it easier to activate past memories through associative processing and make decisions based on intuitive thinking when faced with clear and specific information with high interpretability than when faced with abstract and vague information with low interpretability. As a result, this leads to a greater sense of uncertainty, triggers negative emotions, and consequently elicits larger P2 amplitudes. Hence, this study proposes the following hypotheses.

#### H3

For privacy empowerment illusion cues with low comprehensibility, high interpretability induces a greater P2 amplitude than does low interpretability.

#### H4

For privacy empowerment illusion cues with high comprehensibility, there is no significant difference between the P2 amplitudes induced by high- and low interpretability cues.

N2 is a negative waveform that reaches its peak between 200 and 350 ms after the presentation of a stimulus and is the second negative component that appears after the presentation of a stimulus; this component is primarily located in the frontal, fronto-central, and central areas. N2 originates from the process of conscious cognitive processing and reflects psychological processes such as cognitive conflicts, cognitive control, conflict monitoring, and response inhibition during decision-making tasks. Research has suggested that N2 is associated with cognitive conflicts and plays a significant role in cognitive and behavioural decision making. The N2 component serves as an indicator of task difficulty and individual cognitive effort. N2 is particularly sensitive to conflict detection, with more severe conflict situations inducing greater N2 amplitudes^59^. Researchers investigating product sales and review ratings have found that products with lower user ratings and sales tend to elicit larger N2 amplitudes, indicating a conflict between the situation and the psychological expectations held by users through propositional reasoning^60^. According to behavioural decision-making studies, greater cognitive conflicts induced during decision making result in greater N2 amplitudes^61^. However, when presented with privacy empowerment illusion cues possessing high comprehensibility, individuals hold clear attitudes and do not base their decisions on situational cues, resulting in no difference between the N2 amplitudes induced by high- and low-interpretability cues. In contrast, when presented with privacy empowerment illusion cues possessing low comprehensibility, individuals struggle to extract relevant information directly from situational cues. They use high- and low-interpretability cues to automatically activate related memories. Through an associative emotional initiation strategy, individuals enter the rational judgement processing system and form evaluative judgements based on propositional processing and deductive reasoning. In other words, individuals facing abstract and vague low-interpretability information engage in more propositional processing and deductive reasoning, consuming more cognitive resources than individuals facing specific high-interpretability information. This leads to greater discrepancies between their independently derived ground-truth values and their beliefs and values, resulting in more severe cognitive conflicts and larger N2 amplitudes. Therefore, the following hypotheses are proposed.

#### H5

For privacy empowerment illusion cues with low comprehensibility, low interpretability induces a greater N2 amplitude than does high interpretability.

#### H6

For privacy empowerment illusion cues with high comprehensibility, there is no significant difference between the N2 amplitudes induced by high- and low-interpretability cues.

**Figure 1:**
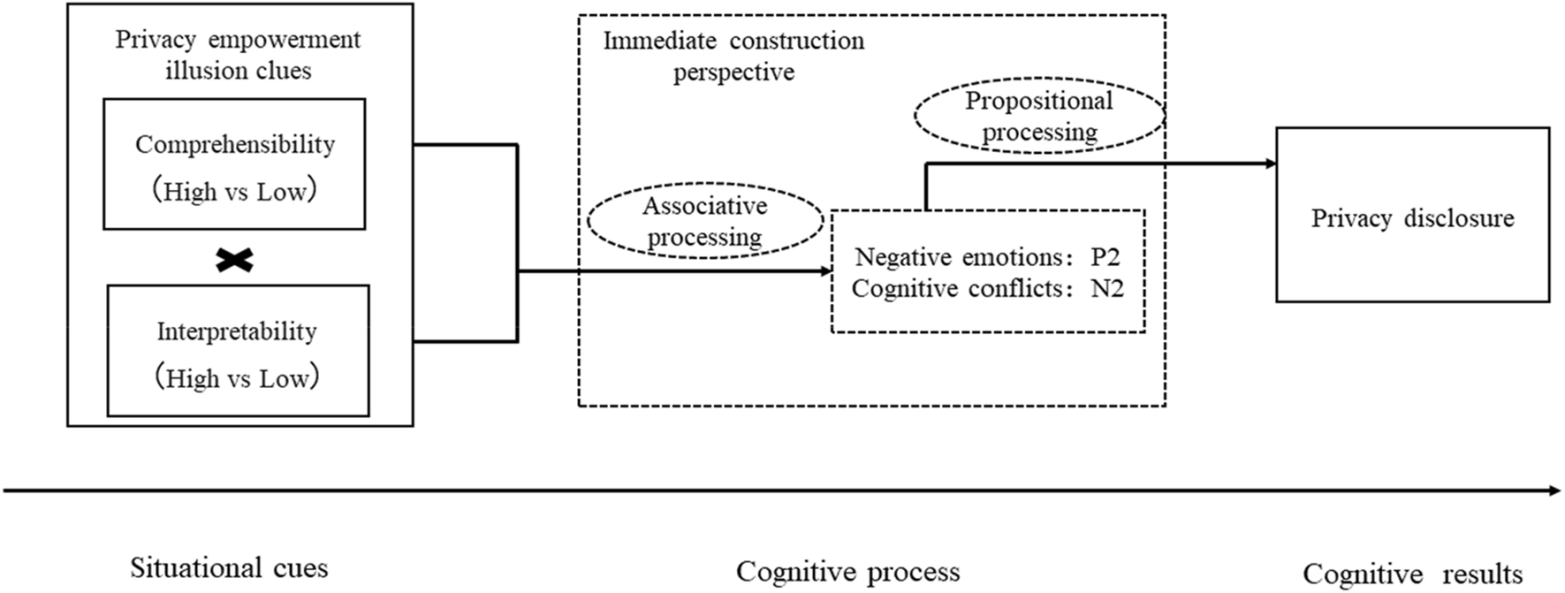
Research model.

The theoretical model of this study is shown in Fig. 1.

## Experiments

### Subjects

Based on the objectives and design of this study, the recruited subjects needed to be divided into privacy empowerment illusion condition groups with high and low comprehensibility. The operational details are as follows. This research identified the most prevalent privacy empowerment illusion cue, namely, the privacy statement. The participants we recruited were evenly divided into two groups, with each group consisting of equal numbers of males and females. For the participants in the privacy empowerment illusion condition with high comprehensibility, an event report that gives users the illusion of privacy empowerment was shared with them for 15 consecutive days before conducting the formal experiment, alongside succinct, lucid, comprehensible materials highlighting the perils of privacy empowerment illusions; for example, “When a user downloads a certain shopping app, the app will display a privacy policy pop-up, asking them to choose ‘agree’ or ‘refuse’. If they choose ‘refuse’, they can no longer use the shopping app and must click agree, which severely deprives users of their choice” and “After installing a certain health tracking app, users find that refusing personal data collection means they cannot track their workouts and that ‘agree to share’ has been set as the ‘default’ choice.” Moreover, for the low-comprehensibility group, the experimental materials consisted of reports on privacy empowerment illusion incidents filled with technical terminology, obscure language, and generalized statements that are hard to comprehend. For instance, "Certain apps utilize catch-all provisions during information collection, featuring phrases such as 'including but not limited to,’ ‘obtaining additional user information for XXX's needs,’ and ‘related information, etc.,’ to gather and use user data" and "The updated privacy policy of a specific music streaming service mentions using ‘LBS technology’ to ‘improve user experience,’ thereby enabling prolonged tracking of user GPS location data”. After conducting the experiment, the users’ understanding of privacy empowerment illusion incidents was assessed through direct interviews.

This mixed-design study considered the comprehensibility (high vs. low) and interpretability (high vs. low) of information as the grouping criteria. The interpretability of privacy empowerment illusion cues served as a within-group variable, while comprehensibility served as a between-group variable. Following Cohen’s standards, we calculated the required sample size for this study using G*power 3.1^62,63^. The calculated minimum total sample size was 24 participants. A recent systematic review of the applicability of the scales employed in consumer neuroscience studies revealed that the average sample size used in previous research ranged from 16 to 42^64,65^. Consequently, we recruited 26 participants (13 males and 13 females) with an average age of 22.33 years, satisfying the required sample size for the experiment. All participants were right-handed, had no history of mental illness, and possessed normal unaided or corrected vision (participants with myopia were required to bring their own framed eyeglasses to ensure proper vision correction and prevent visual fatigue caused by the prolonged use of contact lenses). Before beginning the formal experiment, all participants signed informed consent forms. During the experiment, participants had the option to stop at any time if they experienced physical discomfort. Regardless of whether the experiment was completed, the participants received compensation as appropriate.

### Experimental materials

#### Experimental scenario

To ensure that the participants clearly understood the privacy empowerment illusion context, this study selected 30 commonly occurring instances of privacy empowerment illusion phenomena as alternative experimental materials. Fifty nonparticipants (the individuals involved in the manipulative test were excluded from participating in the EEG experiment to prevent familiarity effects) were randomly selected to view these materials and answer the following question: “To what extent do you consider this event to be a privacy empowerment illusion event?” A seven-point Likert scale ranging from 1 (strongly disagree/not applicable) to 7 (strongly agree/applicable) was used for the survey. The 16 materials with the highest scores were selected for the formal experiment. The formal experiment included but was not limited to the following scenarios.

a. On certain platforms, the use of certain features requires facial recognition authentication. When users are asked for permission, if they decline, they cannot continue to use that feature or even other features.

b. On some platforms, although users are given the option to disable personalized ad recommendations, the process involves 12 complex steps, can be disabled for only six months, and results in only a partial reduction in the preference relevance level rather than entirely unrelated generic ads.

The interpretability of privacy empowerment illusion cues. Given the inherent limitations of neuroscience experiments, the situations and materials used must be as simple and clear as possible. This research employed the most concise language to describe the common scenario of information usage transparency. The privacy empowerment illusion cues with high interpretability included “We will obtain your approximate location information through the local area network connected to your mobile device” and “We will appropriately provide personalized services based on your past browsing records,” while the privacy empowerment illusion cues with low interpretability included “To facilitate end users to identify the geographical location of social sharing, we will collect information from end users, including geographical location, by calling system-related interfaces” and “We establish user preference vectors and product rating vectors based on collaborative filtering algorithms and make recommendations accordingly.”

## Experimental procedure

**Figure 2:**
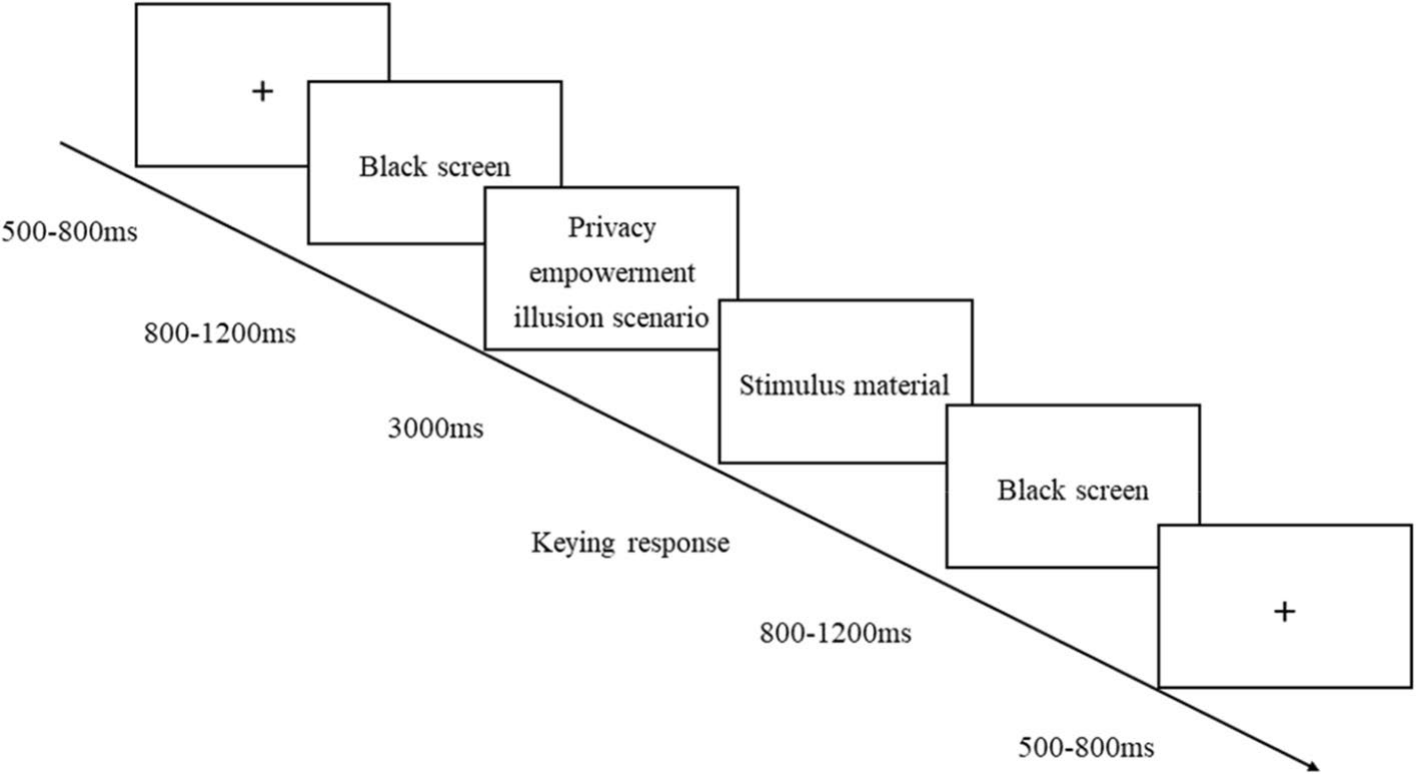
Experimental flowchart.

Before the experiment began, the participants were instructed to carefully read the experimental instructions, which were supplemented by further explanations from the experimenter, to ensure that the participants correctly understood the experimental procedures and could complete the experiment effectively. The specific experimental procedure was as follows: first, a privacy empowerment illusion scenario was presented (the “space” key was pressed to enter the decision interface after reading about the scenario); second, in the decision interface, explorable privacy empowerment illusion cues were presented (high vs. low). The participants were asked to decide whether they were willing to continue with the privacy authorization decision on this platform based on the presented cues (pressing the “F” key to indicate agreement and pressing the “J” key to indicate refusal). The detailed experimental procedure is illustrated in Fig. 2.

The experiment consisted of two stages—practice and the formal experiment—for a total of 108 trials. The practice stage included 12 trials, while the formal experiment was divided into 2 blocks with different scenarios, with each block comprising 48 trials. To eliminate sequence effects, different stimulus materials were randomly presented in each trial. During the experiment, the participants were asked to focus their attention and minimize behaviours such as blinking, swallowing saliva, and making large movements.

### Institutional review board statement

The study was conducted in accordance with the Declaration of Helsinki and approved by the Ethics Committee of Huaqiao University (M2023009 2023.4.19).

### Informed consent statement

Informed consent was obtained from all the subjects involved in the study.

## Results

### Behavioural data

Independent-sample t tests were conducted to compare the privacy disclosure rates between the participants in different comprehensibility groups. The results revealed a significant difference between the privacy disclosure rates of the participants in the privacy empowerment illusion group with high comprehensibility (M = 0.301, SD = 0.155) and those in the privacy empowerment illusion group with low comprehensibility (M = 0.676, SD = 0.158), t(24) =  − 8.295, p = 0.000 < 0.001. Thus, the comprehensibility grouping of privacy empowerment illusion cues was successful.

Privacy empowerment illusion cues with low comprehensibility: Independent-sample t tests were conducted to compare the privacy disclosure rates of the participants in the low-comprehensibility group when exposed to different interpretability cues (high vs. low). The results showed that the privacy disclosure rate for the privacy empowerment illusion cues with high interpretability (M = 0.775, SD = 0.065) was significantly greater than that of the privacy empowerment illusion cues with low interpretability (M = 0.577, SD = 0.163), t(12) = 3.927, p = 0.001 < 0.01. Thus, H1 was supported.

Privacy empowerment illusion cues with high comprehensibility: Independent-sample t tests were conducted to compare the privacy disclosure rates of participants in the high-interpretability group when exposed to different interpretability cues (high vs. low). The results showed no significant differences between the privacy disclosure rates of the high-interpretability (M = 0.348, SD = 0.148) and low-interpretability privacy empowerment illusion cues (M = 0.255, SD = 0.154), t(12) = 1.501, p = 0.148 > 0.050. Thus, H2 was supported.

### EEG data

Based on grand-averaged butterfly plots, the P2 component was analysed within a time window of 180–240 ms using electrode sites CP3, CPZ, and CP4 in the parietal region. The N2 component was analysed within a 220–280 ms window using electrode sites F1, FZ, and F2 in the central brain region. Repeated-measures analysis of variance (ANOVA) was also conducted on the amplitudes of the P2 and N2 components.

### P2 component results

**Figure 3:**
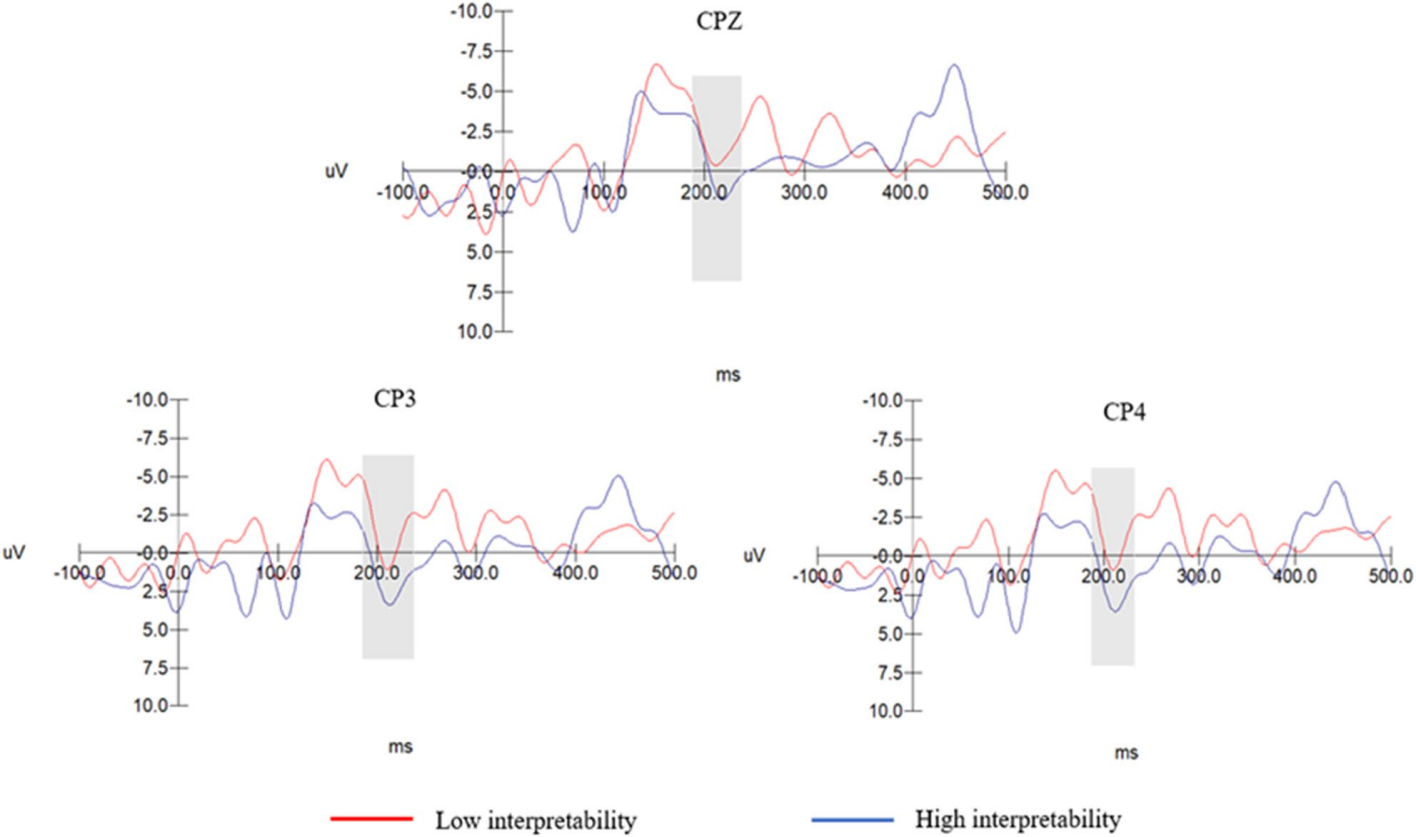
P2 component amplitudes of privacy empowerment illusion cues with high and low interpretability levels under low comprehensibility.

In the low-comprehensibility privacy empowerment illusion group, the main effect of the electrode point was significant (F (2, 44) = 16.957, p = 0.000 < 0.001), and the interpretable interaction effect between the electrode point and privacy empowerment illusion cues was significant (F (2, 44) = 4.241, p = 0.028 < 0.050). The main effect of the interpretability of the privacy empowerment illusion cues was significant (F (1, 24) = 25.78, p = 0.000 < 0.001). According to the estimated marginal mean, the P2 component of the privacy empowerment illusion cues with low interpretability (M = 2.659, SD = 1.374) was significantly lower than that of the privacy empowerment illusion cues with high interpretability (M = 12.527, SD = 1.374). As privacy empowerment illusion cues become increasingly clear, participants tend to experience more negative emotions. Thus, H3 was supported. See Fig. 3 for more details.

Privacy empowerment illusion cues with high comprehensibility: The electrode sites exhibited a significant main effect (F(2, 44) = 10.847, p = 0.000 < 0.001). The interaction effect between the electrode sites and the interpretability of the privacy empowerment illusion cues was not significant (F(2, 44) = 0.911, p = 0.409 > 0.050). The main effect of the interpretability of the privacy empowerment illusion cues was also not significant (F(1, 24) = 0.000, p = 0.986 > 0.05). Thus, H4 was supported.

### N2 component results

**Figure 4:**
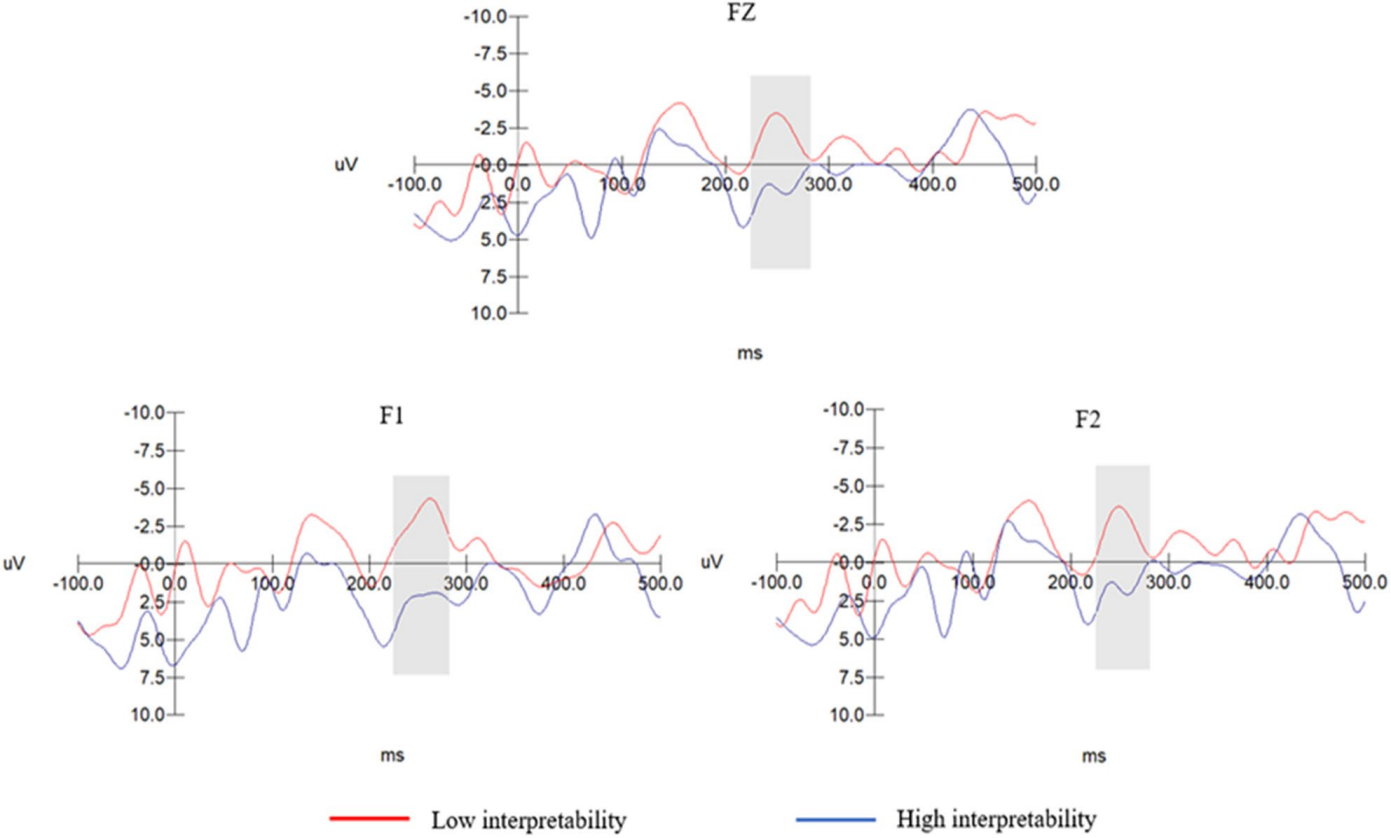
N2 component amplitudes obtained for privacy empowerment illusion cues with high and low interpretability under low comprehensibility.

Privacy empowerment illusion cues with low comprehensibility: The electrode sites did not exhibit a significant main effect (F(2, 44) = 1.975, p = 0.151 > 0.050). Additionally, no significant interaction effect was observed between the electrode sites and the interpretability of the privacy empowerment illusion cues (F(2, 44) = 0.073, p = 0.929 < 0.010). The main effect of the interpretability of the privacy empowerment illusion cues was not significant (F(1, 24) = 0.001, p = 0.976 > 0.05). Thus, H6 was supported. No significant main effect was yielded by the electrode sites (F(2, 44) = 0.148, p = 0.863 > 0.050). However, a significant interaction effect was observed between the electrode sites and the interpretability of the privacy empowerment illusion cues, F(2, 44) = 4.291, p = 0.027 < 0.050. The main effect of the interpretability of the privacy empowerment illusion cues was significant (F(1, 24) = 48.159, p = 0.000 < 0.001). Based on the estimated marginal means, the N2 component amplitude in the privacy empowerment illusion cues with high interpretability (M =  − 0.202, SD = 0.430) was significantly lower than that in the cues with low interpretability (M =  − 4.421, SD = 0.430). Thus, H5 was supported. Figure 4 shows the associated details.

Privacy empowerment illusion cues with high comprehensibility: No significant main effects were exhibited by the electrode sites (F(2, 44) = 1.975, p = 0.151 > 0.050). Additionally, no significant interaction effect was observed between the electrode sites and the interpretability of the privacy empowerment illusion cues (F(2, 44) = 0.073, p = 0.929 < 0.010). The main effect of the interpretability of the privacy empowerment illusion cues was not significant (F(1, 24) = 0.001, p = 0.976 > 0.05). Thus, H6 was supported.

## Discussion

### Conclusions

In the era of internet monopolies, the “empowerment process” of digital platforms can never truly become an “equalization process”. Platforms merely create an illusion of empowerment through privacy empowerment illusion tactics, covertly stripping users of control and data sovereignty, exploiting consumer surpluses, and infringing upon user privacy. However, users have minimal concerns about the privacy risks associated with privacy empowerment illusions and rarely implement privacy protection measures. The paradox of privacy empowerment is a hot topic in current privacy research. This study, based on the APE model and cognitive load theory, examined the inherent impacts of the comprehensibility and interpretability of privacy empowerment illusion cues on users' cognitive loads and immediate attitudes, constructing a research model for privacy disclosure. Utilizing ERP techniques to obtain neurophysiological indicators from consumers in the form of EEG data, this research aimed to understand users' underlying cognitive patterns and the impacts of these patterns on their immediate privacy attitudes and behavioural decisions. Based on the experimental research, this study draws the following conclusions.

1. User privacy disclosure behaviour differences are produced based on interpretability. Specifically, regarding the privacy empowerment illusion cues with low comprehensibility, users were more willing to disclose their private information in response to cues with high interpretability than in response to cues with low interpretability. However, for the privacy empowerment illusion cues with high comprehensibility, no significant difference was observed between the user privacy disclosure behaviours exhibited in response to high- and low interpretability cues.
2. Among the privacy empowerment illusion cues with low comprehensibility, the cues with low interpretability led to more negative emotions in individuals than did the cues with high interpretability, eliciting larger P2 amplitudes. That is, in the privacy empowerment illusion cues with low comprehensibility, abstract and vague privacy explanations induced more negative emotions in users, leading to larger P2 wave amplitudes. However, for the privacy empowerment illusion cues with high comprehensibility, no significant difference was observed between the P2 amplitudes elicited by high- and low interpretability cues.
3. When utilizing the privacy empowerment illusion cues with low comprehensibility, individuals encountered more cognitive conflicts and expended more cognitive resources in response to low-interpretability cues than in response to high-interpretability cues, resulting in larger N2 amplitudes. That is, among the privacy empowerment illusion cues with low comprehensibility, abstract and vague explanatory cues depleted more cognitive resources in individuals, leading to larger N2 wave amplitudes. However, for the privacy empowerment illusion cues with high comprehensibility, the N2 amplitudes elicited by high-and low-interpretability cues were not significantly different.

### Theoretical contributions

This study makes the following theoretical contributions.

First, it explores the impact of privacy empowerment illusion cues on user privacy disclosure from the perspectives of immediate constructions and cognitive loads, providing new insights into both research areas. In the field of privacy empowerment, scholars have often focused on discussing of newly emerged privacy empowerment illusion cues, primarily the vagueness, abstraction, comprehensibility, and readability of information cues in relation to behavioural decision making^28,31^. Moreover, many studies have concentrated on general and holistic evaluations of users^5,32^. However, privacy information in reality is specific and concrete, and users’ privacy attitudes, which determine their behaviours, are constructed in real time through cognitive and emotional reactions. Therefore, this study, based on the perspectives of the immediate constructions and cognitive loads of users, selects comprehensibility and interpretability cues in the context of privacy empowerment illusions and clarifies the impacts of immediate privacy attitudes constructed through the effects of automatic emotional responses and cognitive loads for interpretability cues with different levels of comprehensibility on behavioural decisions. This study provides insights into users' real cognitive load states and immediate privacy attitudes, aligning with the actual online decision-making context and users’ intrinsic emotional and cognitive patterns, thus offering a fresh research perspective for determining on the impact of privacy empowerment illusion cues on user privacy disclosure.

Second, this study extends the scope of research on the privacy empowerment paradox based on the APE model and cognitive load theory. Previous studies have frequently analysed privacy attitudes from a rational user viewpoint, resulting in the establishment of dominant explanatory frameworks such as privacy calculus theory, communication privacy management theory, and privacy cynicism^7,9–11^. These theories suggest that behavioural decisions are the outcomes of users' rational comprehensive assessments of risks and benefits. However, the privacy attitudes addressed in the existing research are holistic perceptions formed through retrospective scenarios^11^, leaving a “black box” regarding the differing psychological processes that users undergo when assessing benefits in immediate situations; this study also examines the impacts of genuine, instantaneously constructed privacy attitudes on behaviour decisions made during this processing phase. The existing research paradigms are constrained by the “hypothetical response in a hypothetical situation” scenario, and the decisions made through retrospective whole-brain processing do not correspond to users' actual behaviours. In reality, individuals make immediate decisions about privacy disclosures that are influenced largely by their emotions and cognitive loads. Therefore, this study leverages the APE model and cognitive load theory to elucidate users’ cognitive processes in the context of privacy empowerment illusion cues. It delves into how comprehensibility and interpretability cues trigger different psychological processes (associative processing and propositional processing) and constructs real-time privacy attitudes that influence privacy decisions based on the cognitive resources expended during unconscious emotional activation and conscious reasoning. This research expands the applicability of the APE model and cognitive load theory in the field of the privacy empowerment paradox.

Finally, this study employs neuroscientific techniques to offer a novel approach through which future experimental research can analyse the real-time construction of user cognitive states and privacy attitudes in the context of privacy empowerment illusions. Traditional research methods, such as interviews and behavioural measurements, are valuable for conducting extensive studies^45,66^. However, researchers often struggle to obtain real-time insights into users’ cognitive load states and the way in which they construct their immediate privacy attitudes. From a neurophysiological perspective, this study uses EEG signal analysis to investigate the underlying mechanisms through which privacy empowerment illusion cues influence users’ cognitive loads and their privacy attitudes constructed in real time^67^. By analysing specific psychological phenomena through real-time physiological electrical signal indicators, this approach provides more accurate measurements of users' cognitive and emotional responses, including different psychological processes such as associative processing and propositional processing. Directly observing certain latent variables from individual brain signals and measuring immediate cognitive and emotional responses effectively reduce the potential biases associated with the subjective data used in traditional research methods^67^. This approach allows researchers to delve deeper into the “black box” of the psychological processes performed by users under cognitive loads and their emotional responses.

### Practical significance

The privacy issues addressed in this study stem from real-life online contexts. By investigating the underlying mechanisms of the privacy empowerment paradox and understanding the factors that influence users’ privacy decision-making processes, this research contributes to the field of privacy protection. It offers insights that can guide digital enterprises and platforms towards more feasible privacy protection paths. Collaboration among various stakeholders can be fostered to create a more reliable and secure online environment, facilitating the free and comprehensive development of personalized technologies. Based on the comprehensive empirical analysis results presented, this study proposes the following privacy protection strategies and recommendations for safeguarding the information of individuals.

#### User perspective: enhancing users’ literacy concerning online privacy

The advancement of the internet has brought about changes in not only connectivity but also the transfer and diffusion of power and identity information among various entities. To a certain extent, users act as “passive” consumers of the internet and should proactively learn how they are attracted to and “captivated” by the online world. Only by continually raising awareness of algorithms and privacy can individuals reduce or mitigate the risks associated with information disclosure. A lack of online privacy literacy may lead to a failure to promptly and accurately perceive and evaluate the issues that are related to privacy attitudes and behaviours. This can inadvertently empower those with malicious intents to exploit private information, resulting in more severe privacy breaches.

On the one hand, it is essential to cultivate users’ ability to see through the essence of the internet and the potential hazards of technology dissemination. In the context of the new internet landscape, instances of privacy empowerment illusion are rampant and increasingly “covert,” often ensnaring users into algorithmic spirals from which they cannot easily extricate themselves. Therefore, it is crucial to increase users’ knowledge of internet media and improve their technical skills. In addition to mastering the necessary internet skills, users should expand their understanding of internet media; maintain a discerning mindset; improve their ability to discern the authenticity of information; and develop the capacity to share, create, and disseminate the knowledge contained in internet media. This will result in individuals forming a profound awareness and judgements of the online environment, allowing them to objectively assess both the progress and risks brought about by technology. This, in turn, will help prevent excessive panic or blind indulgence despite the consequences of technology. On the other hand, users must recognize their own capabilities in terms of handling privacy issues and avoiding excessive optimism, which could lead to the leakage of personal information. Platforms may manipulate users through the illusion of control, coaxing them into disclosing more private information based on the superficial perception of control. Therefore, users need to have a clear understanding of their ability to control their information and take proactive steps to safeguard the security of their personal information. This will help prevent unnecessary encroachments on their data rights resulting from the excessive disclosure of private information.

#### Platform perspective: improving the comprehensibility and interpretability of privacy empowerment cues and fostering ethical awareness among technical controllers

Addressing the issue of black-box algorithms from a technical standpoint is relatively effective. This necessitates a clear understanding of the fundamental sources of user discomfort caused by black-box algorithms, enabling improvements to be made in the design stage to prevent the rise of privacy concerns caused by the mishandling of technology. When individuals cannot control how their private information is collected and used, their privacy concerns are heightened. However, the demands of users' social existences require them to disclose necessary personal information, which is precisely why this paradox arises.

On the one hand, privacy empowerment should ensure fairness, comprehensibility and interpretability to protect users' personal information. Companies should cease embedding their own biased interests into the privacy empowerment process and treat each user's information equally, avoiding practices such as price discrimination based on big data. Additionally, they should provide the public with explanations of the decision-making processes of their algorithms, ensuring users' fundamental right to be informed. This study revealed that different levels of interpretability lead to different immediate attitudes, with high-interpretability cues helping mitigate overall negative evaluations of privacy empowerment. Therefore, platforms should, to the greatest extent possible, use specific, easily readable, and clear language to inform users of how they collect, process, and employ user information, granting users reasonable informed consent. On the other hand, those who control the technology experience privacy empowerment. The current privacy crisis in online society is fundamentally a crisis of control between individuals. Utilizing private data as a medium, the personal information of ordinary users is controlled and used by corporations and capitalists. This kind of control and usage can not only lead to the leakage of private information but also potentially trap individuals in an information silo, ultimately causing a loss of value. When algorithmic programs are misused, algorithmic bias occurs. Therefore, to harness algorithms without bias, it is necessary to increase the ethical awareness of algorithm designers, urge them to collect and use personal information ethically and properly, and avoid the undue manipulation of users through algorithms. This would serve as a constraint on algorithms as well as on platforms and capitalists.

### Limitations and future prospects

Through the construction of a theoretical model and corresponding empirical research, this study provides a systematic and in-depth analysis of the privacy empowerment paradox, yielding novel findings. This study has certain limitations and calls for additional comprehensive and in-depth research to be conducted in the future. First, this study explores the impacts of individual instant privacy attitudes such as emotional activations and cognitive conflicts on privacy disclosure in online contexts. However, privacy empowerment is rich in content and multidimensional in structure. Different individuals may exhibit varying attitude responses and behavioural outcomes in response to privacy empowerment illusion tactics, intentions, consequences, and other factors. Subsequent research should attempt to refine the contexts of privacy empowerment illusion and investigate the relationships among emotional activation, cognitive conflict, and immediate decision making in various contexts. Second, the impact of privacy empowerment illusion on the disclosure of users’ private information is influenced not only by the comprehensibility and interpretability of cues but also by personal factors such as the level of involvement, the degree of harm, and the bandwagon effect, as well as by considerations of social interactions such as social norms and cultural influences. Furthermore, as the sample population of this study comprised college students, it did not account for individual literacy levels, which have more substantial practical effects on research outcomes. Hence, future studies should incorporate factors such as the level of involvement, the degree of harm, the conformity effect, literacy, and social norms to delve deeper into their inherent effects on users' instant attitudes and behaviours. Third, the experiment enrolled college students as participants, which may limit the external validity of the study. However, college students often have higher internet usage frequencies and skills than other demographic groups, which might expose them to more instances of privacy empowerment illusion. Moreover, the participants came from various regions across the country and had diverse cultural backgrounds and perspectives, which contributed to sample diversity and helped mitigate sampling errors. Additionally, the study collected data in a controlled laboratory setting, which allowed for better control of extraneous variables and the examination of direct stimulus‒response relationships. However, this setting may not completely simulate real-world situations, introducing some level of bias. Therefore, future research should consider conducting related behavioural experiments to address these limitations.

## Data Availability

The data are available upon request due to privacy or ethical restrictions. The data presented in this study are available upon request from the corresponding author.
